# Potential intervention targets to promote physical activity among people with multiple sclerosis: A scoping review protocol for evidence of moderation

**DOI:** 10.1371/journal.pone.0351882

**Published:** 2026-06-23

**Authors:** Emily R. Jakob, Steve Amireault, Jason B. Reed, Shih-Chun Kao, Robert W. Motl, Elizabeth A. Richards, Reese C. Kerschner

**Affiliations:** 1 Department of Health & Kinesiology, Purdue University, West Lafayette, Indiana, United States of America; 2 Libraries and School of Informational Studies, Purdue University, West Lafayette, Indiana, United States of America; 3 Department of Kinesiology and Nutrition, University of Illinois Chicago, Chicago, Illinois, United States of America; 4 School of Nursing, Purdue University, West Lafayette, Indiana, United States of America; 5 Department of Psychological Sciences, Purdue University, West Lafayette, Indiana, United States of America; PLOS: Public Library of Science, UNITED KINGDOM OF GREAT BRITAIN AND NORTHERN IRELAND

## Abstract

**Introduction:**

Although currently available behavioral interventions can be effective at promoting physical activity among people with multiple sclerosis (MS), there is inconsistency in the magnitude of reported intervention effects. This highlights the importance of exploring the available evidence of moderation, which can help inform decisions about whether a specific set of behavior change techniques and strategies should be adopted for different contexts or segments of the population. Therefore, we propose a scoping review protocol to guide a summary of the body of work that has attempted to understand, explain, predict, or change physical activity behavior (context) among people with MS (population), with an eye on evidence of moderation (concept).

**Methods and analyses:**

Studies that report on a sample of people with MS in the context of physical activity will be included for review. The search strategy includes the combing of eight electronic databases, a hand search of the reference lists for all included studies and relevant knowledge syntheses, and a citing reference search for all included studies using Web of Science. Two reviewers will single-screen titles and abstracts and independently assess each study based on a full text review. Types of multiple sclerosis populations included, and types of research methodologies used in the studies will be presented using a waffle chart. Results pertaining to the evidence of moderation will be presented using a tree graph.

**Dissemination:**

Results will be published in a peer-review journal.

## Introduction

Multiple sclerosis (MS) is an immune-mediated, neurodegenerative disorder of the central nervous system that results in the destruction of the myelin sheath of nerve cells, also known as demyelination [1]. Diagnosis of MS usually occurs in people aged 20–50 years old and is most prevalent in adults, however, children can also be affected by the disorder [2,3]. People with MS live a full life expectancy but experience symptoms that vary in prevalence and severity across different people. Some of the most often cited symptoms of MS are physical and cognitive functional limitations, gait impairments, anxiety and depression, and fatigue [4]. The course of MS varies, but the most common course is Relapsing Remitting MS in which people experience week-to-month-long periods of symptoms, called “relapse”, coupled with week-to-month-long periods of partial or full recovery called “remission” [4].

Physical activity is considered as one of the most effective strategies for managing MS-related symptoms such as fatigue, depression, cognitive impairments, balance and walking abilities, and mitigating MS progression [5–7]. Consistent with this body of evidence, the National MS Society recommends physical activity as a as a safe and effective addition to medication for management of symptoms and modification of the disease [8–11]. However, physical activity participation typically declines after people received a diagnosis of MS [12–14]. Although currently available behavioral interventions can be effective at promoting physical activity among people with MS, there is inconsistency in the magnitude of reported intervention effects [15–20]. In fact, the magnitude of effects vary substantially across experimental studies (standardized mean differences ranged from 0.03 to 1.42 for immediate post-interventions effects, and 0.17 to 1.08 for sustained 12-week follow-up effects [19], with some providing supportive evidence and others providing insufficient evidence for the effectiveness of interventions to change physical activity [15,19,20]). This inconsistency, often referred to as heterogeneity, in the magnitude of effects within this body of work lowers the certainty of evidence and strength of recommendations for practices [21]. Collectively, these observations highlight the need for investigators and clinicians to examine factors that could explain the variability in the magnitude of behavioral intervention effectiveness at increasing and maintaining physical activity participation among people with MS.

### Translational framework

A fundamental first step in developing behavioral interventions that are maximally effective at increasing and maintaining physical activity behavior among people with MS is the identification of factors that are related to that behavior and potentially malleable [22–26]. Such factors qualify as high-priority targets for behavioral interventions. Thereafter, the specification of intervention targets orients the selection of behavior change techniques and strategies towards those that are precisely aimed at activating or changing the specified targets. Therefore, these factors, when specified as intervention targets, represent a set of potential mechanisms of action through which an intervention is expected to impact physical activity behavior. From this perspective, behavioral interventions that include components engaging all the appropriate mechanisms of actions in the warranted direction are more likely to be maximally effective at promoting physical activity than those who do not.

Consistent with this perspective, the Operating Conditions Framework [27] emphasizes the importance of identifying specific contexts and segments of the population for which the specified intervention targets for behavior change are more or less effective. To this aim, the gathering of evidence of moderation can enable investigators and clinicians to specify when and for whom intervention targets are more or less effective at increasing or maintaining physical activity behavior. As such, a knowledge base with a focus on evidence of moderation can help inform decisions about whether a specific set of behavior change techniques and strategies should be adopted for different contexts or segments of the population.

**Fig 1:**
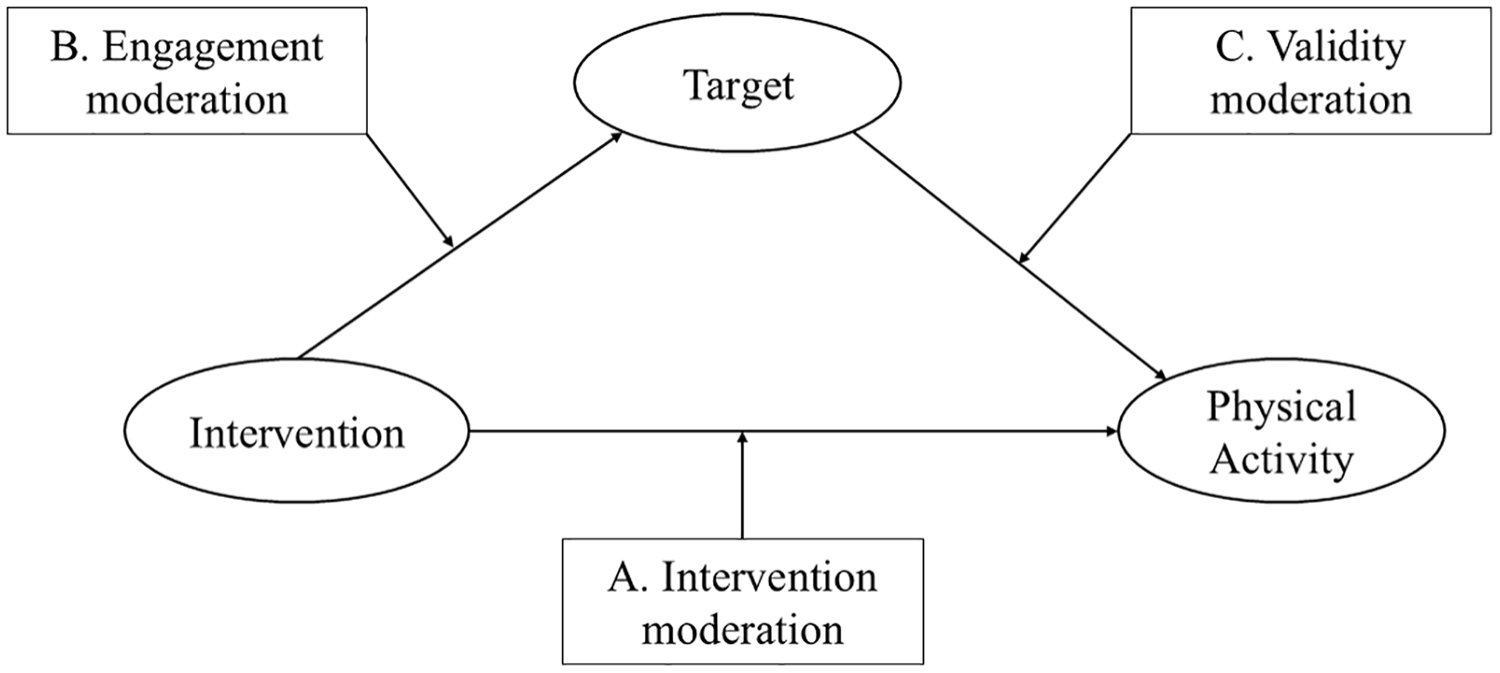
Moderators of the impact of behavioral interventions on physical activity behavior. *Note*. Panel A depicts evidence of moderation that identifies the conditions under which a behavioral intervention is more or less effective at changing or maintaining physical activity behavior. Intervention moderation examines whether intervention effectiveness differs across individuals or contexts. Panel B depicts evidence of moderation that identifies the conditions under which a behavioral intervention is more or less effective at engaging a specific intervention target. Engagement moderation examines whether an intervention differs in its ability to influence a specific intervention target across individuals or contexts. Panel C depicts evidence of moderation that identifies conditions under which an intervention target is more or less effective at changing or maintaining physical activity behavior. Validity moderation examines whether the effect of an intervention target on physical activity behavior differs across individuals or contexts.

Investigators and clinicians often rely on health behavior theories and studies identifying correlates or determinants of behaviors for specifying targets for a behavioral intervention. However, health behavior theories rarely specify the conditions under which the constructs (i.e., potential intervention targets) operate [26]. Further, moderator analyses in empirical studies of people with MS [15–20] are often limited on testing moderators of the total effect of an intervention on physical activity behavior among people with MS (Fig 1; Panel A). As depicted in Fig 1, such an approach is suboptimal because it is unlikely to capture the precise explanation that underlies this evidence of moderation. On one hand, the behavioral intervention may work best (or might not work) because the intervention targets are appropriate across contexts or segments of the population, but a different set of behavior change techniques or strategies is needed in specific contexts or segments of the population for the behavioral intervention to be optimally successful (Fig 1; Panel B). On the other hand, the behavioral intervention may work best (or might not work) because the intervention is effective at eliciting favorable change in the intervention targets uniformly across contexts or segments of the population, but one or more of the intervention targets are less effective for some contexts or segments of the population (Fig 1; Panel B). That is, evidence of moderation for the total effect of an intervention on physical activity behavior among people with MS may indicate that (*i*) one or more behavioral techniques or strategies were more or less effective at changing the intervention targets for some contexts or segments of the population, (*ii*) one or more intervention targets were more or less effective in some contexts or segments of the population, (*iii*) or both.

Herein, we propose a scoping review protocol to guide a summary of the body of work that has attempted to understand, explain, predict, or change physical activity behavior among people with MS, with an eye on evidence of moderation. In the context of the proposed scoping review, the concept of moderation represents the variation in the degree to which a given factor (i.e., potential target for interventions) relates to physical activity behavior as a function of another factor, called a moderator variable. Specifically, a moderator would be a variable that changes the direction (sign) or magnitude (size) of the relation between a factor that may qualify as a target for intervention interventions and physical activity behavior. We restrict this proposed scoping review to this above type of moderation evidence, also described as validity moderation [27], because the specification of potential intervention targets represents a fundamental first step in the creation of behavioral interventions that have the potential to be maximally effective at promoting physical activity [22,26,27]. The proposed knowledge synthesis work is exploratory in nature. That is, the overall objective is to examine what has been studied and what remains unexamined regarding the evidence of moderation of the relations between factors that may qualify as a target for interventions and physical activity behavior among people with MS. Because the focus is on the identification of the characteristics of relevant studies and the mapping of evidence that pertains to moderation, a scoping review framework was considered the most appropriate approach for this knowledge synthesis.

We expect the findings of the proposed scoping review to make a significant contribution to the health and physical activity psychology literature within the MS population by identifying key characteristics of relevant studies, mapping evidence that pertains to moderation, and highlighting research gaps. As a result, we expect the findings of the proposed scoping review will help encourage and inform the design and conduct of more robust and sensitive moderation analyses. It is also expected that these findings will help investigators to provide a more compelling rationale for the a priori planning and conduct of subgroup and meta-regression analyses of future meta-analyses of studies pertaining to physical activity behavior among people with MS. Ultimately, developing a knowledge base with a focus on evidence of moderation is expected to provide new insights that could help investigators and clinicians identify with greater precision the mechanistic targets of their interventions for promoting physical activity behavior among people with MS, and inform decisions about whether specific set of behavior change techniques and strategies should be adopted for different contexts or segments of that population.

### Scoping review objectives

Following the Population, Concept, and Context acronym (PCC), the primary research question of the proposed scoping review is: What literature exists on evidence of moderation (concept) of the relations between factors that may qualify as a target for interventions and physical activity behavior (context) among people with MS (population)? We propose two sub questions:

1. What range of evidence is there within the sources of evidence identified for the primary research question with respect to the research methods and selected attributes of the research design?
2. Which factors have been identified as a moderator within the sources of evidence identified for the primary research question?

## Methods and analysis

This scoping review protocol was developed following guidance of the scoping review framework proposed by the JBI Manual for Evidence Synthesis [28,29]. The reporting of this scoping review protocol follows the reporting guidelines for scoping review protocols [28], and is consistent with the Preferred Reporting Items for Systematic Review and Meta-Analysis Protocols (PRISMA-P; [30]). An adapted version of the PRISMA-ScR checklist based on the reporting guidelines for scoping review protocols [28] is provided in Appendix A.

The Purdue University Human Research Protection Program determined that that proposed scoping review does not qualify as human subject research under Federal Human Subjects Research Regulations (IRB-2024–1037). We will conduct the proposed scoping review in accordance with the scoping review framework outlined by the JBI Manual for Evidence Synthesis [29,31]. The reporting of the proposed scoping review will follow the Preferred Reporting Items for Systematic Reviews and Meta-Analyses Extension for Scoping Reviews (PRISMA-ScR [32]). Any deviations to the protocol, along with their respective justification, will be reported in the final scoping review report.

### Eligibility criteria

#### Participants.

Studies that report on a sample of people with MS will be included for review. Participants with any form of MS (i.e., primary progressive, relapsing-remitting, or secondary progressive) will be considered for inclusion. Studies involving samples that include recreational or professional athletes, individuals with no diagnosis of MS, or individuals who were unable to engage in physical activity, will be excluded from the review.

#### Concepts.

Table 1 lists the operational definitions of PCC components for the proposed scoping review.

**Table 1:** Definitions of PCC components for the proposed scoping review.

PCC components	Definition
Physical activity	Any human movement produced by the contraction of skeletal muscles that raises energy expenditure above resting metabolic rate (i.e., 1 Metabolic Equivalent of Task; MET). Physical activity is inclusive of the term exercise and sport. Exercise refers to physical activity that is planned, structured, and repetitive for the purpose of enhancing or maintaining physical fitness and health. Sport refers to physical activity that is rule governed, structured, competitive, and involves gross motor movement characterized by physical strategy, prowess, and chance. Unstructured physical activity that is unplanned and done for purposes outside of maintaining fitness will also be included. Physical activity is not inclusive of the concept sedentary behaviors, which refer to any waking behaviors characterized by an energy expenditure ≤ 1.5 METs, while in a sitting, reclining, or lying posture.
Multiple Sclerosis	Multiple Sclerosis (MS) is a neurovegetative disorder that presents a variety of symptoms and degrees of severity. Some of the most common symptoms are motor impairments, fatigue, anxiety, depression, and pain. There are three possible courses of the disorder, including relapsing-remitting MS (patients experience periods of symptoms that can last weeks to month, coupled with similarly-lengthened periods of remission in which symptoms subside or disappear entirely), secondary-progressive MS (following relapsing-remitting MS, when periods of remission are no longer present and MS symptoms worsen as the disorder progresses), primary-progressive MS (progression of the disorder and worsening of symptoms occur at time of diagnosis and no periods of remission are ever present). For this review, studies involving any form of MS will be considered.
Validity moderation	Moderation represents the variation in the degree to which a factor that may qualify as an intervention targets relate to physical activity as a function of another factor, called a moderator or effect modifier variable. Moderators specify characteristics of the population (for whom?) or conditions (when or where?) the relation between a given factor that may qualify as targets for interventions is more or less strongly related to physical activity behavior.

*Note*. PCC: Population, Concept, and Context.

#### Types of evidence sources.

The proposed scoping review will draw upon data from studies that have used a qualitative, quantitative, or mixed methodological approach. We will include both peer-reviewed journal articles and theses and dissertations. We will include studies that have used cross-sectional, longitudinal, quasi-experimental and experimental design. Knowledge synthesis of any types, conference abstracts, commentaries, editorials, study protocols, books and book chapters, and case studies will be excluded.

### Search strategy

The search strategy was developed by the review team, which includes a health sciences librarian and content experts in kinesiology, physical activity psychology, public health, and MS in the context of physical activity. The health sciences librarian implemented and executed an initial electronic databases (coverage period) search strategy for CINAHL (EBSCO interface; 1976 – present), EMBASE (Elsevier interface; 1947 – present), APA PsycInfo (EBSCO interface; 1887 – present), PubMed (1946 – present), SPORTDiscus (EBSCO interface; 1930 – present), SciELO (scielo.org; 2002 – present), VHL Regional Portal (bvsalud.org; 1998 – present), and Dissertations and Theses (ProQuest interface; 1938 – present; includes Dissertations & Theses @ Big Ten Academic Alliance; ProQuest Dissertations & Theses Global; ProQuest Dissertations & Theses Global Closed Collection). For all databases, search terms taping the MS population and physical activity concept were used. Because MS diagnosis typically occurs in adults between the ages of 20 and 50 years old but can also, less commonly, be diagnosed in children and adolescents [2,3], no age filters were used for any of the databases. Additionally, we used database-specific Index or Medical Subject Headings (MeSH) terms when available. The free text search terms remained constant across all databases, searching across title, abstract, and when available, keyword fields. We updated the database-specific terms for each database, where available, but used the same key concepts across all the databases. We used filters for resource types (i.e., publication type article, article in press, conference paper, conference review, review, preprints) in EMBASE. No date or language filters were used. Full details of the search strategy are provided in Appendix B.

To identify additional studies, two reviewers (ERJ, SA) will independently perform a hand search of the reference lists for all included studies and relevant knowledge syntheses [33–36]. Lastly, we will conduct a citing reference search for all included studies using Web of Science. The results of the search will be reported in a PRISMA-ScR flow-chart [32]. Although knowledge syntheses were excluded, we ran an additional search using this strategy but limited results to knowledge syntheses. We did this to confirm that no other knowledge syntheses on this topic exist.

Studies written in any language will be included. Title and abstract of any potential studies that are reported in a language other than English will be initially translated using Google Translate. The full text will be translated by a qualified translator if it meets the inclusion criteria at that stage. It is worth noting that at least one member of the review team has high proficiency in English, French, and Mandarin. Lastly, we will place no constraints on the publishing year of the studies. We will use Covidence, a web-based software platform, to manage the retrieved citation records (Covidence systematic review software, Veritas Health Innovation, Melbourne, Australia).

### Source of evidence selection

We ran an initial electronic databases search on 5/6/2024, which yielded a total of 8712 records (*k*), after removing duplicates. We will not use any automation tools for study selection.

#### Screening of titles and abstracts.

Given the high number of identified records through our initial electronic citation search (*k* = 8712) and the related time and cost constraints associated with their screening, two reviewers (ERJ, RCK) will single-screen titles and abstracts using an over-inclusive approach. Specifically, a given record will be retained and considered for full-text examination if there is insufficient information to conclude with certainty its exclusion. The specific exclusion criteria considered at this stage were conference abstract, review article, case report/series, not human research, ineligible populations, and not reporting on the concept of MS and the concept of physical activity. We pilot-tested the titles and abstracts screening procedure using a random sample of 107 records from our initial search which were independently screened for eligibility by two review members (SA, ERJ). The decisions were compared, and discrepancies among the two reviewers were resolved by discussion. At the end of this step, the reviewers disagreed on three studies, and both reviewers agreed to exclude them after discussion. Then one of the reviewers involved in the first step described above met with one other reviewer (RCK) to provide training and discuss screening decisions. Upon pilot-testing the process of screening titles and abstract, and given the number of records to screen, the review team decided that all titles and abstracts would be screened by a single reviewer, one of the two involved in pilot-testing titles and abstracts (ERJ) and the additional trained reviewer (RCK). The single screening of the titles and abstracts can yield high sensitivity (98% − 100%) when certain exclusion criteria are used such as conference abstract, review article, case report/series, non-human research, and it is acceptable at this stage of the selection process [37,38]. Screening of titles and abstracts began in June of 2024 and was completed in November of 2024.

#### Full-text examination.

We will retrieve the full text of the records selected for inclusion at the titles and abstracts screening stage. Two reviewers will independently assess the eligibility of each article. Multiple articles reporting on the same study and written by the same authors group will be gathered and scrutinized to ensure that only unique study, rather than each article or duplication study, represents the unit of interest in the scoping review. When necessary for the making of a selection decision, we will contact the authors of the articles for unpublished information. The decisions to include an article will be compared between the two reviewers, and discrepancies between reviewers will be resolved by discussion. When no consensus can be reached, a third reviewer will help resolve the discrepancy. Moreover, any relevant retraction statements and errata for information for each included article will be examined to exclude data from studies that are fraudulent or studies that include errors. We will report reasons for exclusion of full-text articles that do not meet the eligibility criteria in a supplementary PRISMA flowchart in the scoping review.

### Data extraction

Prior to data extraction, two reviewers will independently pilot-test a purpose-built data extraction sheet with three randomly selected records. An initial version of the purpose-built data extraction sheet is provided in Appendix C. More specifically, reviewers will extract relevant information regarding all types of physical activity – all physical activity measurement metrics (e.g., frequency, duration, intensity, number of steps, arbitrary activity units) will be considered, as well as the measurement by means of device-based (e.g., accelerometers) and self-report (e.g., questionnaires) instruments. We will extract information on factors linked to physical activity behavior. For each study included, we will collect observations and findings pertaining to evidence of moderation. We will identify if a moderating factor was investigated (yes/no), the results pertaining to moderation, and the main conclusion of the study. We will also specify the theoretical approach underlying the investigation (if any), and the methodological approaches for investigating moderating effects (e.g., subgroup analysis or formal statistical test of interactions).

It is expected that the data extraction sheet will be refined and revised following the pilot-testing of the data extraction process. After pilot-testing of the data extraction sheet, we will hold a meeting with all scoping review authors to discuss all aspects of the revised version of the data extraction sheet and agree on its final version. Then, the reviewers will independently extract data from all included studies, compare results, and resolve any discrepancies through discussion. We will contact the study authors for clarification on unreported data item. When no consensus on reported data items can be reached, we will contact (maximum of three attempts) the corresponding author of the study to help resolve the discrepancy. We will hold bi-weekly meetings throughout the data extraction process to discuss progress and monitor whether the data extraction sheet is capturing all the essential information to properly answer the research questions.

### Data analysis and presentation of results

Data analysis will primarily be descriptive, reporting frequency counts and percentages. Primary study and sample characteristics will be reported for descriptive purposes in a summary table. We will use a waffle chart to visually present the different types and number of MS populations included within the primary studies (i.e., relapsing-remitting MS, primary progressive MS, and secondary progressive MS) and indicate the types of research methodologies used (i.e., qualitative, quantitative, and mixed methods) by the primary studies.

Prior to mapping the evidence of moderation, the review authors will familiarize themselves with the data by reading and understanding all the included studies for review and understanding the relevance of the data in relation to the main scoping review question and sub questions. We will analyze the data descriptively, and report frequency count and percentage of studies investigating and reporting on evidence of moderation. Results of this qualitative synthesis will be presented using a tree graph to display the moderating factor(s) investigated by the included studies, and a ratio indicating the number of time evidence for moderation was reported by the total number of investigations. It is worth noting that this reporting is intended only to point out potential moderating factors to consider in future primary studies (i.e., thus serving a hypothesis-generating function) rather than specifying a given documented factor as a moderator (i.e., serving a hypothesis-testing function). This mapping will be applied to all studies, irrespective of their research methods or context for physical activity.

We will perform a narrative synthesis of the findings to highlight similarities and differences both within and across primary studies by examining the convergence and divergence in findings across qualitative, quantitative, and mixed-methods methodological approach, concept of physical activity, and approaches for investigating moderating effects.

### Dissemination plan

The results of the proposed scoping review will be published in a peer-reviewed journal and presented at relevant scientific conferences.

### Registration

The protocol for this proposed scoping review has been registered with the Open Science Framework (OSF; Jakob et al., 2026; osf.io/4tepu).

## Supporting information

S1 Appendix Preferred Reporting Items for Systematic reviews and Meta-Analyses extension for Scoping Reviews (PRISMA-ScR) Checklist adapted for scoping review protocol. (DOCX)

S2 Appendix Search Strategy—MS and Exercise—Run 5/6/24. (DOCX)

S3 Appendix Purpose-built data extraction sheet. (DOCX)
