# Supplementary material for: Potential intervention targets to promote physical activity among people with multiple sclerosis: A scoping review protocol for evidence of moderation
Source: PLoS One. 2026 Jun 23;21(6):e0351882. doi: 10.1371/journal.pone.0351882 (PMC13289874; doi:10.1371/journal.pone.0351882)
Supplement: S2 Appendix — (DOCX) [file pone.0351882.s002.docx]

Appendix B: Search Strategy

Date of the search: May 6^th^, 2024

| PubMed | No filters applied  Results: 4,612  1. "Multiple Sclerosis"[Mesh] 2. Multiple Sclerosis[Title/Abstract] 3. #1 OR #2 4. "Exercise"[MeSH Terms] OR "Physical Fitness"[MeSH Terms] 5. "exercis*"[Title/Abstract] OR "physical activity"[Title/Abstract] OR "physical fitness"[Title/Abstract] OR "walking"[Title/Abstract] OR "walked"[Title/Abstract] OR "walk"[Title/Abstract] 6. #4 OR #5 7. #3 AND #6 8. "animals"[MeSH Terms] NOT "humans"[MeSH Terms] 9. #7 NOT #8 |
| --- | --- |
| EMBASE | Filters: Publication type article, article in press, conference paper, conference review, review, preprints (included in search strategy)  Results: 6,124  1. 'multiple sclerosis'/exp 2. 'multiple sclerosis':ti,ab,kw 3. #1 OR #2 4. 'exercise'/exp OR 'physical activity'/exp 5. 'exercis*':ti,ab,kw OR 'physical activity':ti,ab,kw OR 'physical fitness':ti,ab,kw OR 'walking':ti,ab,kw OR 'walked':ti,ab,kw OR 'walk':ti,ab,kw 6. #4 OR #5 7. #3 AND #6 8. #7 NOT ('animals'/exp NOT 'humans'/exp) 9. #3 AND #6 AND ([article]/lim OR [article in press]/lim OR [conference paper]/lim OR [conference review]/lim OR [review]/lim OR [preprint]/lim) |
| APA PsycInfo | No filters  Results: 1,175  1. DE "Multiple Sclerosis" 2. TI multiple sclerosis OR AB multiple sclerosis OR SU multiple sclerosis  3. S1 OR S2 4. DE "Exercise" OR DE "Aerobic Exercise" OR DE "Weightlifting" OR DE "Yoga" OR DE "Physical Activity" OR DE "Actigraphy" OR DE "Walking" 5. TI ( "exercis*" OR "physical activity" OR "physical fitness" OR "walking" OR "walked" OR "walk" ) OR AB ( "exercis*" OR "physical activity" OR "physical fitness" OR "walking" OR "walked" OR "walk" ) OR SU ( "exercis*" OR "physical activity" OR "physical fitness" OR "walking" OR "walked" OR "walk" )  6. S4 OR S5 7. S3 AND S6 |
| SPORTDiscus | No filters applied  Results: 1,257  1. DE "MULTIPLE sclerosis" 2. TI multiple sclerosis OR AB multiple sclerosis OR KW multiple sclerosis  3. S1 OR S2  4. DE "PHYSICAL activity" OR DE "EXERCISE" OR DE "ABDOMINAL exercises" OR DE "AEROBIC exercises" OR DE "ANAEROBIC exercises" OR DE "AQUATIC exercises" OR DE "ARM exercises" OR DE "BACK exercises" OR DE "BREATHING exercises" OR DE "BREEMA" OR DE "BUTTOCKS exercises" OR DE "CALISTHENICS" OR DE "CHAIR exercises" OR DE "CHEST exercises" OR DE "CIRCUIT training" OR DE "COMPOUND exercises" OR DE "COOLDOWN" OR DE "DO-in" OR DE "EXERCISE adherence" OR DE "EXERCISE for children" OR DE "EXERCISE for girls" OR DE "EXERCISE for men" OR DE "EXERCISE for middle-aged persons" OR DE "EXERCISE for older people" OR DE "EXERCISE for people with disabilities" OR DE "EXERCISE for women" OR DE "EXERCISE for youth" OR DE "EXERCISE therapy" OR DE "EXERCISE video games" OR DE "FACIAL exercises" OR DE "FALUN gong exercises" OR DE "FOOT exercises" OR DE "GYMNASTICS" OR DE "HAND exercises" OR DE "HATHA yoga" OR DE "HIP exercises" OR DE "ISOKINETIC exercise" OR DE "ISOLATION exercises" OR DE "ISOMETRIC exercise" OR DE "ISOTONIC exercise" OR DE "KNEE exercises" OR DE "LEG exercises" OR DE "LIANGONG" OR DE "METABOLIC equivalent" OR DE "MULAN quan" OR DE "MUSCLE strength" OR DE "PILATES method" OR DE "PLYOMETRICS" OR DE "QI gong" OR DE "REDUCING exercises" OR DE "RUNNING" OR DE "SCHOOL exercises & recreations" OR DE "SEXUAL exercises" OR DE "SHOULDER exercises" OR DE "STRENGTH training" OR DE "STRESS management exercises" OR DE "TAI chi" OR DE "TREADMILL exercise" OR DE "WHEELCHAIR workouts" OR DE "YOGA" OR DE "WALKING" OR DE "FITNESS walking" OR DE "GAIT in humans" OR DE "HIKING" OR DE "LONG distance walking" OR DE "VIERDAAGSE (Walking event)" OR DE "PHYSICAL fitness" 5. TI ( "exercis*" OR "physical activity" OR "physical fitness" OR "walking" OR "walked" OR "walk" ) OR AB ( "exercis*" OR "physical activity" OR "physical fitness" OR "walking" OR "walked" OR "walk" ) OR KW ( "exercis*" OR "physical activity" OR "physical fitness" OR "walking" OR "walked" OR "walk" )  6. S4 OR S5 7. S3 AND S6 |
| Dissertations and Theses (ProQuest) includes [ Dissertations & Theses @ Big Ten Academic Alliance; ProQuest Dissertations & Theses Global; ProQuest Dissertations & Theses Global Closed Collection] | No Filters  Results: 329  1. title(multiple sclerosis ) OR summary(multiple sclerosis ) OR subject(multiple sclerosis ) 2. title("exercis*" OR "physical activity" OR "physical fitness" OR "walking" OR "walked" OR "walk") OR summary("exercis*" OR "physical activity" OR "physical fitness" OR "walking" OR "walked" OR "walk") OR subject("exercis*" OR "physical activity" OR "physical fitness" OR "walking" OR "walked" OR "walk") 3. [S1] AND [S2] VHL Regional Portal  Filtered to the collections: LILACS, IBECS, WPRIM, BIGG, Index Psychology journals, medRxiv, Coleciona SIS, AIM (Africa), BDENF – Nursing, BDNPAR, BINACIS, BRISA/RedTESA, PIE Results: 109 ( mh:("Exercise" AND "Multiple Sclerosis")) OR (multiple sclerosis) AND (exercise* OR "physical activity" OR "physical fitness" OR "walking" OR "walked" OR "walk") ((( mh:(“Exercise”)) OR (exercise* OR "physical activity" OR "physical fitness" OR "walking" OR "walked" OR "walk")) AND (( mh:("Multiple Sclerosis")) OR (multiple sclerosis))) |
| SciELO | No filters  Results: 20  (“Multiple sclerosis) AND ("exercis*" OR "physical activity" OR "physical fitness" OR "walking" OR "walked" OR "walk") |
| CINAHL | No filters  Results: 2,722   1. (MH "Multiple Sclerosis+") 2. TI multiple sclerosis OR AB multiple sclerosis OR MW Multiple Sclerosis 3. S1 OR S2 4. (MH "Exercise+") OR (MH "Physical Activity") OR (MH "Physical Fitness+") 5. TI ( "exercis*" OR "physical activity" OR "physical fitness" OR "walking" OR "walked" OR "walk" ) OR AB ( "exercis*" OR "physical activity" OR "physical fitness" OR "walking" OR "walked" OR "walk" ) OR MW ( "exercis*" OR "physical activity" OR "physical fitness" OR "walking" OR "walked" OR "walk" ) 6. S4 OR S5 7. S3 AND S6 |
| VHL Regional Portal | Filtered to the collections: LILACS, IBECS, WPRIM, BIGG, Index Psychology journals, medRxiv, Coleciona SIS, AIM (Africa), BDENF – Nursing, BDNPAR, BINACIS, BRISA/RedTESA, PIE  Results: 109  (mh:("Exercise" AND "Multiple Sclerosis")) OR (multiple sclerosis) AND (exercise* OR "physical activity" OR "physical fitness" OR "walking" OR "walked" OR "walk") |
