# Supplementary material for: Potential intervention targets to promote physical activity among people with multiple sclerosis: A scoping review protocol for evidence of moderation
Source: PLoS One. 2026 Jun 23;21(6):e0351882. doi: 10.1371/journal.pone.0351882 (PMC13289874; doi:10.1371/journal.pone.0351882)
Supplement: S3 Appendix — (DOCX) [file pone.0351882.s003.docx]

Appendix C: Purpose-built data extraction sheet

| **Characteristics of the study** |
| --- |
| Authors |
| publication year |
| country of origin |
| Aim |
| **Population and sample characteristics** |
| sample size  mean age  stage of MS  mean time since diagnosis  mean disability level  demographic information |
| Whether the analysis is confirmatory (pre-specified) or exploratory (post hoc) in nature. |
| **Methodological approach**  Qualitative (through differential qualitative analysis or comparative subgroup analysis)  Quantitative, through statistical test for interaction or subgroup analysis |
| **Type of study design:** cross-sectional, longitudinal, quasi-experimental, or experimental |
| **Physical activity measurement metrics** |
| Frequency  Duration  Volume  Arbitrary activity units |
| **Physical activity method of measurement** |
| device-based |
| self-report |
| **Theoretical framework** |
| reporting of one theory or framework reported (yes/no) |
|  |
| **Evidence of moderation** |
| a moderating factor was investigated (yes/no)  number of moderation analyses conducted (i.e., whether zero, one or several moderation analyses were reported and conducted). |
| methodological approaches for investigating moderating effects  reporting by the authors of statistical power estimation for the moderation analyses |
| results pertaining to moderation |
| **Data analysis and statistical reporting**  Information pertaining to the distribution (i.e., score variability and range) of the variables  Independent variables (i.e., factors that may qualify as a target for intervention)  Moderator variables  Whether a subgroup or interaction effect was detected, along with their corresponding statistical information [for quantitative moderation analysis only] |
| **Main conclusion of the study**  Reporting by the author(s) of the primary studies of whether the evidence of moderation, or lack of thereof, is plausible, and provide justification for this judgement.  Reporting by the author(s) of the primary studies of whether the evidence of moderation, or lack of thereof, is a theoretically or clinically important finding, and provide justification for this judgement.  Reporting by the author(s) of the primary studies of whether any other factors were identified that might be confounded with the moderator variable of interest in the moderation analyses. |
|  |
