# Supplementary material for: Potential intervention targets to promote physical activity among people with multiple sclerosis: A scoping review protocol for evidence of moderation
Source: PLoS One. 2026 Jun 23;21(6):e0351882. doi: 10.1371/journal.pone.0351882 (PMC13289874; doi:10.1371/journal.pone.0351882)
Supplement: Attachment — Submitted filename: Description of Revisions-1.31.2025-FINAL.docx [file pone.0351882.s004.docx]

**Description of Revisions/Responses to Reviewers’ Comments**

**PLOS ONE - Manuscript ID PONE-D-24-43698**

**"Potential intervention targets to promote physical activity among people with multiple sclerosis: A scoping review protocol for evidence of moderation”**

We thank the Editor and the reviewers for their careful reading and constructive criticism of the manuscript.

**Editor(s)' Comments to Author (if any):**

My decision is in line with reviewers and I will ask you to do a major revision.

Response: Our responses to each of the specific comments of the reviewers (italicized) are described below. The revision and the ensuing modifications are marked in the main text using a red font color.

**Reviewer #1:**

***Comments to the Authors:***

*Thank you for the opportunity to review this manuscript*.‎

1. *Could the authors elaborate on why a scoping review is deemed more appropriate for ‎addressing the research question compared to a systematic review?‎*

Response #1: We thank the reviewer for this comment. The proposed knowledge synthesis work is exploratory in nature. That is, the overall objective is to examine what has been studied and what remains unexamined regarding the evidence of moderation of the relations between factors that may qualify as a target for interventions and physical activity behavior among people with multiple sclerosis (MS). Because the focus is on the identification of the characteristics (including the conduct and reporting) of relevant studies and the mapping of evidence that pertains to moderation, we considered the scoping review framework to be the most suitable approach for achieving the overall objective of our proposed knowledge synthesis. We added this information to the manuscript on pages 6-7, lines 159-166

2. *How do the authors plan to ensure that all relevant studies are identified and included? ‎For example, is the use of seven databases and hand-searching sufficient to capture the ‎breadth of the literature?‎*

Response #2: We thank the reviewer for this question. The electronic search strategy consists of the searching of relevant documents in eight databases, including the two main biomedical databases that Cochrane recommends for systematic review searches (PubMed and EMBASE), an physical activity-specific database (SPORTDiscus), a psychology-specific database (APA PsycInfo) and two regional specific databases that are recommended for capturing potential evidence that is not indexed in the major resources. This diversity of databases is intended to ensure that relevant studies are identified for screening. The search strategy also includes a combination of controlled search vocabularies (such as MeSH) and key terms searched in title/abstract fields to further ensure that relevant studies are identified. Lastly, the hand searching component will provide a check for any potential studies missed in the database search. These approaches are following the best guidance for conducting evidence synthesis, in terms of the breadth and quantity of databases included, supplemented with hand searching.

3. *How will the authors address potential biases or inconsistencies in the reporting of ‎moderating factors across the included studies?‎*

Response #3: We thank to reviewer for this comment. Variability in the reporting of moderating factors across the study included will be a key piece of information we intend to collect to identify potential research gaps. Based on the work of and recommendations by Bloom & Michalopoulos (2013), Frazier et al. (2004) and Richardson et al. (2019), the following data items will be extracted and collected from each primary study:

1- Methodological approach:
   a. Qualitative (through differential qualitative analysis or comparative subgroup analysis)
   b. Quantitative, through statistical test for interaction or subgroup analysis
2- Whether the analysis is confirmatory (pre-specified) or exploratory (post hoc) in nature.
3- Reporting of the number of moderation analyses conducted (i.e., whether zero, one or several moderation analyses were reported and conducted).
4- Reporting by the authors of statistical power estimation for the moderation analyses.
5- The reporting by the author(s) of the primary studies of information pertaining to the distribution (i.e., score variability and range) of the independent (i.e., factors that may qualify as a target for intervention) and moderator variables.
6- The reporting by the author(s) of the primary studies of whether a subgroup or interaction effect was detected, along with their corresponding statistical information [for quantitative moderation analysis only].
7- Reporting by the author(s) of the primary studies of whether the evidence of moderation, or lack of thereof, is plausible, and whether they provided justification for this judgement.
8- Reporting by the author(s) of the primary studies of whether the evidence of moderation, or lack of thereof, is a theoretically or clinically important finding, and whether they provide justification for this judgement.
9- Reporting by the author(s) of the primary studies of whether any other factors were identified that might be confounded with the moderator variable of interest in the moderation analyses.

This information has been added to the data item extraction sheet.

**References**:

Bloom, H. S., & Michalopoulos, C. (2013). When is the story in the subgroups? Strategies for interpreting and reporting intervention effects for subgroups. Prevention Science, 14(2), 179–188. https://doi.org/10.1007/S11121-010-0198-X

Frazier, P. A., Tix, A. P., & Barron, K. E. (2004). Testing moderator and mediator effects in counseling psychology research. Journal of Counseling Psychology, 51(1), 115–134. https://doi.org/10.1037/0022-0167.51.1.115

Richardson, M., Garner, P., & Donegan, S. (2019). Interpretation of subgroup analyses in systematic reviews: A tutorial. Clinical Epidemiology and Global Health, 7(2), 192–198. https://doi.org/10.1016/J.CEGH.2018.05.005

4. *Could the authors clarify how the proposed ratio (number of times moderation is reported ‎vs. total investigations) will be interpreted? Additionally, how will studies with ‎incomplete or ambiguous reporting of moderation factors be handled?‎*

Response #4: As indicated in manuscript (pages 16; lines 320-323 and lines 334-337), the reporting of frequency counts and percentages will primarily serve a descriptive purpose. This information is intended only to point out potential moderating factors to consider in future primary studies (i.e., serving a hypothesis-generating function) rather than specifying a given documented factor as a moderator (i.e., serving a hypothesis-testing function).

**Reviewer: 2**
***Comments to the Author:***

*Dear author/s,*

*Thank you for the opportunity to review this manuscript, which presents a protocol for a scoping review on promoting physical activity (PPA) among people with multiple sclerosis (MS). The protocol is well presented and provides a framework for an interesting and useful review. The search strategy, which includes the PRISMA-ScR checklist, appears to be comprehensive.*

5. *My only concern is ensuring that the review does not extend beyond the limits of a scoping review. The research questions regarding evidence of moderation of the relations between factors that may qualify as a target for interventions and PPA among people with MS may lead to different synthesis methods. A scoping review will allow you to map these features as they are reported in the literature, but it is not an appropriate method for providing evidence on effectiveness or etiology. If the main goal of the review is to map and characterize features of the reported literature on PPA among MS, then a scoping review is appropriate. However, I would suggest rephrasing the research questions slightly to align with the acronym PCC, which is more common for scoping reviews - Population, Concept, Context - as recommended in reference 27: Peters, M., Godfrey, C., McInerney, P., Khalil, H., Larsen, P., Marnie, C., Pollock, D., Tricco, A., Munn, Z. Best practice guidance and reporting items for the development of scoping review protocols. JBI Evidence Synthesis 20(4): p 953-968, April 2022. | DOI: 10.11124/JBIES-21-00242.*

*If, however, the main goal is to determine effectiveness, then I suggest using systematic review methods, which will allow you to determine effectiveness, or a review of etiology and risk for identifying causal factors influencing outcomes.

These articles may be of use:*

*Munn, Z., Peters, M.D.J., Stern, C. et al. Systematic review or scoping review? Guidance for authors when choosing between a systematic or scoping review approach. BMC Med Res Methodol 18, 143 (2018). https://doi.org/10.1186/s12874- 018-0611-x*

*Tricco, A.C., Lillie, E., Zarin, W. et al. A scoping review on the conduct and reporting of scoping reviews. BMC Med Res Methodol 16, 15 (2016). https://doi.org/10.1186/s12874-016-0116-4*

Response #5: We thank the reviewer for this comment. As stated in Response #1, The proposed knowledge synthesis work is exploratory in nature. That is, the overall objective is to examine what has been studied and what remains unexamined regarding the evidence of moderation of the relations between factors that may qualify as a target for interventions and physical activity behavior among people with MS. Because the focus is on the identification of the characteristics (including the conduct and reporting) of relevant studies and the mapping of evidence that pertains to moderation, we considered the scoping review framework to be the most suitable approach for achieving the overall objective of our proposed knowledge synthesis. We added this information to the manuscript on pages 6-7, lines 157-164.

As suggested by this reviewer, we now present the primary scoping review question in the manuscript by specifying the PCC acronym (Page 7, line 180-184).

*However, the feedback provided outlines several critical areas for improvement in the manuscript before it can be accepted for publication. Addressing these points will enhance the clarity and quality of the research presented.*

6. *The author should rewrite the abstract, because it does not adequately describe your work. The authors should ensure that it accurately reflects the scope and outcomes of their study.*

Response #6: We have made changes to the abstract to reflect comments and proposed edits from reviewers to ensure that it accurately reflects the scope and outcomes of their study

7. *The introduction is pivotal in setting the stage for the research. It should clearly articulate the main ideas and motivations behind the proposed study. The authors must explicitly state the research question and the motivation for their work, ensuring that the research gap is adequately addressed. This clarity will help readers understand the context and importance of the research.*

Response #7: Thank you for this comment. The primary research question and two sub questions are stated in the Scoping Review Objectives section of the manuscript (pages 7-8, lines 180-190). The main ideas and motivations (rationale) are articulated in the Translational Framework sections of the manuscript (pages 3-6, lines 80-138).

8. *Add a table of used symbols in the paper to improve readability.*

Response #8: This manuscript does not contain symbols outside of grammatical symbols. We have omitted the use of abbreviations for the different courses of MS (i.e. RRMS, SPMS, and PPMS) and instead spelled out the respective courses. The only abbreviation that we now have is MS for multiple sclerosis in substitution of adding a table.

9. *The authors should comment on the relevance of the results, comparison of the approaches, and critical review of the relevant literature.*

Response #9: This manuscript reports a scoping review protocol. According to the JBI Manual for Evidence Synthesis, the key functions of a scoping review protocol are to pre-defines the objectives, methods, and reporting of the review to allow for transparency of the review process and its reporting. As such, we do not comment on the relevance of the results per se. Nonetheless, we have edited and clarified our statement of the expected results of the proposed scoping review (page. 7, lines 167-179).

**Reference**:

Peters MDJ, Godfrey C, McInerney P, Munn Z, Tricco AC, Khalil, H. Scoping Reviews (2020). Aromataris E, Lockwood C, Porritt K, Pilla B, Jordan Z, editors. JBI Manual for Evidence Synthesis. JBI; 2024. Available from: https://synthesismanual.jbi.global. https://doi.org/10.46658/JBIMES-24-09

10. *The authors should identify and discuss open problems related to their research. Highlighting these issues can provide a pathway for future research and demonstrate the ongoing relevance of their work.*

Response #10: According to the JBI Manual for Evidence Synthesis, the key functions of a scoping review protocol are to pre-defines the objectives, methods, and reporting of the review to allow for transparency of the review process and its reporting. Based on the JBI Manual for Evidence Synthesis, a scoping review should include the following components: a title, an introduction, the inclusion criteria, a description of the proposed search strategy, a description of the process of source selection for all stages of selection, a description of the proposed data extraction process, a description of the ways in which the data will be analyzed and synthesized, and a description of a plan for the presentation of results.

A discussion regarding the open problems related to the proposed research (review-level limitations) and of the characteristics (i.e., the conduct and reporting) of relevant primary studies and the mapping of evidence that pertains to moderation (i.e., potential methodological or research gaps) will be reported and discussed in the full scoping review report. See also Response #9. Nonetheless, we acknowledge the following review-level strengths and potential limitation:

- The proposed scoping review will include both qualitative and quantitative studies, providing a more complete picture of the availability of evidence of moderation.
- The electronic database search was designed and pilot-tested to ensure high sensitivity and reliability while balancing time and cost constraints.
- Given that an assessment of the risk of bias of the studies included in the proposed review will not be conducted, the specific or immediate clinical and policy implications of the proposed review may be limited.

**Reference**:

Peters MDJ, Godfrey C, McInerney P, Munn Z, Tricco AC, Khalil, H. Scoping Reviews (2020). Aromataris E, Lockwood C, Porritt K, Pilla B, Jordan Z, editors. JBI Manual for Evidence Synthesis. JBI; 2024. Available from: https://synthesismanual.jbi.global. https://doi.org/10.46658/JBIMES-24-09

11. *The manuscript currently lacks sufficient literature citations. The authors should expand their discussion of related work.*

Response #11: This manuscript reports a scoping review protocol and therefore does not include results or discussion of how these results relate to previous literature. This information, however, will be included in the subsequent final scoping review report (i.e., after the scoping review has been conducted).

12. *It is crucial for authors to address potential biases or inconsistencies when reporting on moderating factors. This involves a systematic approach to ensure that the findings are credible and reflect the true nature of the relationships being studied.*

Response #12: We thank to reviewer for this comment. Variability in the reporting of moderating factors across the study included will be a key piece of information we intend to collect to identify potential research gaps. Based on the work of and recommendations by Bloom & Michalopoulos (2013), Frazier et al. (2004) and Richardson et al. (2019), the following data items will be extracted and collected from each primary study:

10- Methodological approach:
    a. Qualitative (through differential qualitative analysis or comparative subgroup analysis)
    b. Quantitative, through statistical test for interaction or subgroup analysis
11- Whether the analysis is confirmatory (pre-specified) or exploratory (post hoc) in nature.
12- Reporting of the number of moderation analyses conducted (i.e., whether zero, one or several moderation analyses were reported and conducted).
13- Reporting by the authors of statistical power estimation for the moderation analyses.
14- The reporting by the author(s) of the primary studies of information pertaining to the distribution (i.e., score variability and range) of the independent (i.e., factors that may qualify as a target for intervention) and moderator variables.
15- The reporting by the author(s) of the primary studies of whether a subgroup or interaction effect was detected, along with their corresponding statistical information [for quantitative moderation analysis only].
16- Reporting by the author(s) of the primary studies of whether the evidence of moderation, or lack of thereof, is plausible, and whether they provided justification for this judgement.
17- Reporting by the author(s) of the primary studies of whether the evidence of moderation, or lack of thereof, is a theoretically or clinically important finding, and whether they provide justification for this judgement.
18- Reporting by the author(s) of the primary studies of whether any other factors were identified that might be confounded with the moderator variable of interest in the moderation analyses.

This information has been added to the data item extraction sheet.

**References**:

Bloom, H. S., & Michalopoulos, C. (2013). When is the story in the subgroups? Strategies for interpreting and reporting intervention effects for subgroups. Prevention Science, 14(2), 179–188. https://doi.org/10.1007/S11121-010-0198-X

Frazier, P. A., Tix, A. P., & Barron, K. E. (2004). Testing moderator and mediator effects in counseling psychology research. Journal of Counseling Psychology, 51(1), 115–134. https://doi.org/10.1037/0022-0167.51.1.115

Richardson, M., Garner, P., & Donegan, S. (2019). Interpretation of subgroup analyses in systematic reviews: A tutorial. Clinical Epidemiology and Global Health, 7(2), 192–198. https://doi.org/10.1016/J.CEGH.2018.05.005

13. *The authors explain how to interpret the ratio of the number of times moderation is reported versus the total number of studies.*

Response #13: As indicated in Response #4 and in the manuscript (pages 16; lines 320-323; and lines 334-337), the reporting of frequency counts and percentages will primarily serve a descriptive purpose. This information is intended to point out potential moderating factors to consider in future primary studies (i.e., serving a hypothesis-generating function) rather than specifying a given documented factor as a moderator (i.e., serving a hypothesis-testing function).

14. *It seems that using seven databases and manual search to obtain the breadth of literature may not be enough. The comprehensiveness of the literature review is very important. Relying on only a limited number of databases can lead to gaps in the literature, as different databases may index different journals and articles. A systematic approach to literature search often involves the use of multiple databases, including specialized databases related to a particular field of study, to ensure a thorough exploration of available research.*

Response #14*:* We thank the reviewer for their comment. As indicated in Response #2, the electronic search strategy consists of the searching of relevant documents in eight databases, including the two main biomedical databases that Cochrane recommends for systematic review searches (PubMed and EMBASE), an physical activity-specific database (SPORTDiscus), a psychology-specific database (APA PsycInfo) and two regional specific databases that are recommended for capturing potential evidence that is not indexed in the major resources. This diversity of databases is intended to ensure that relevant studies are identified for screening. The search strategy also includes a combination of controlled search vocabularies (such as MeSH) and key terms searched in title/abstract fields to further ensure that relevant studies are identified. Lastly, the hand searching component will provide a check for any potential studies missed in the database search. These approaches are following the best guidance for conducting evidence synthesis, in terms of the breadth and quantity of databases included, supplemented with hand searching.
