# Supplementary material for: Potential intervention targets to promote physical activity among people with multiple sclerosis: A scoping review protocol for evidence of moderation
Source: PLoS One. 2026 Jun 23;21(6):e0351882. doi: 10.1371/journal.pone.0351882 (PMC13289874; doi:10.1371/journal.pone.0351882)
Supplement: Peer review history

# Peer review: Decision Letter 0

**DOI:** 10.1371/journal.pone.0351882.r001

**Authors:** Ardalan Shariat

11 Dec 2024

PONE-D-24-43698

Potential intervention targets to promote physical activity among people with multiple sclerosis: A scoping review protocol for evidence of moderation

PLOS ONE

Dear Dr. Amireault,

Thank you for submitting your manuscript to PLOS ONE. After careful consideration, we feel that it has merit but does not fully meet PLOS ONE’s publication criteria as it currently stands. Therefore, we invite you to submit a revised version of the manuscript that addresses the points raised during the review process.

Please submit your revised manuscript by Jan 24 2025 11:59PM. If you will need more time than this to complete your revisions, please reply to this message or contact the journal office at plosone@plos.org. When you're ready to submit your revision, log on to https://www.editorialmanager.com/pone/ and select the 'Submissions Needing Revision' folder to locate your manuscript file.

Please include the following items when submitting your revised manuscript:

- A rebuttal letter that responds to each point raised by the academic editor and reviewer(s). You should upload this letter as a separate file labeled 'Response to Reviewers'.
- A marked-up copy of your manuscript that highlights changes made to the original version. You should upload this as a separate file labeled 'Revised Manuscript with Track Changes'.
- An unmarked version of your revised paper without tracked changes. You should upload this as a separate file labeled 'Manuscript'.

If you would like to make changes to your financial disclosure, please include your updated statement in your cover letter. Guidelines for resubmitting your figure files are available below the reviewer comments at the end of this letter.

If applicable, we recommend that you deposit your laboratory protocols in protocols.io to enhance the reproducibility of your results. Protocols.io assigns your protocol its own identifier (DOI) so that it can be cited independently in the future. For instructions see: https://journals.plos.org/plosone/s/submission-guidelines#loc-laboratory-protocols. Additionally, PLOS ONE offers an option for publishing peer-reviewed Lab Protocol articles, which describe protocols hosted on protocols.io. Read more information on sharing protocols at https://plos.org/protocols?utm_medium=editorial-email&utm_source=authorletters&utm_campaign=protocols.

We look forward to receiving your revised manuscript.

Kind regards,

Ardalan Shariat, Ph.D, Post-doc

Academic Editor

PLOS ONE

Journal Requirements:

When submitting your revision, we need you to address these additional requirements.

1. Please ensure that your manuscript meets PLOS ONE's style requirements, including those for file naming. The PLOS ONE style templates can be found at https://journals.plos.org/plosone/s/file?id=wjVg/PLOSOne_formatting_sample_main_body.pdf and https://journals.plos.org/plosone/s/file?id=ba62/PLOSOne_formatting_sample_title_authors_affiliations.pdf

**Additional Editor Comments:**

Dear author/s,

My decision is in line with reviewers and I will ask you to do a major revision.

Regards

[Note: HTML markup is below. Please do not edit.]

Reviewers' comments:

Reviewer's Responses to Questions

**Comments to the Author**

1. Does the manuscript provide a valid rationale for the proposed study, with clearly identified and justified research questions?

The research question outlined is expected to address a valid academic problem or topic and contribute to the base of knowledge in the field.

Reviewer #1: Yes

Reviewer #2: Yes

**********

2. Is the protocol technically sound and planned in a manner that will lead to a meaningful outcome and allow testing the stated hypotheses?

The manuscript should describe the methods in sufficient detail to prevent undisclosed flexibility in the experimental procedure or analysis pipeline, including sufficient outcome-neutral conditions (e.g. necessary controls, absence of floor or ceiling effects) to test the proposed hypotheses and a statistical power analysis where applicable. As there may be aspects of the methodology and analysis which can only be refined once the work is undertaken, authors should outline potential assumptions and explicitly describe what aspects of the proposed analyses, if any, are exploratory.

Reviewer #1: Yes

Reviewer #2: Yes

**********

3. Is the methodology feasible and described in sufficient detail to allow the work to be replicable?

Descriptions of methods and materials in the protocol should be reported in sufficient detail for another researcher to reproduce all experiments and analyses. The protocol should describe the appropriate controls, sample size calculations, and replication needed to ensure that the data are robust and reproducible.

Reviewer #1: Yes

Reviewer #2: No

**********

4. Have the authors described where all data underlying the findings will be made available when the study is complete?

The PLOS Data policy requires authors to make all data underlying the findings described in their manuscript fully available without restriction, with rare exception, at the time of publication. The data should be provided as part of the manuscript or its supporting information, or deposited to a public repository. For example, in addition to summary statistics, the data points behind means, medians and variance measures should be available. If there are restrictions on publicly sharing data—e.g. participant privacy or use of data from a third party—those must be specified.

Reviewer #1: Yes

Reviewer #2: No

**********

5. Is the manuscript presented in an intelligible fashion and written in standard English?

PLOS ONE does not copyedit accepted manuscripts, so the language in submitted articles must be clear, correct, and unambiguous. Any typographical or grammatical errors should be corrected at revision, so please note any specific errors here.

Reviewer #1: Yes

Reviewer #2: Yes

**********

6. Review Comments to the Author

Please use the space provided to explain your answers to the questions above and, if applicable, provide comments about issues authors must address before this protocol can be accepted for publication. You may also include additional comments for the author, including concerns about research or publication ethics.

You may also provide optional suggestions and comments to authors that they might find helpful in planning their study.

(Please upload your review as an attachment if it exceeds 20,000 characters)

Reviewer #1: Thank you for the opportunity to review this manuscript.

1.Could the authors elaborate on why a scoping review is deemed more appropriate for addressing the research question compared to a systematic review?

2.How do the authors plan to ensure that all relevant studies are identified and included? For example, is the use of seven databases and hand-searching sufficient to capture the breadth of the literature?

3.How will the authors address potential biases or inconsistencies in the reporting of moderating factors across the included studies?

4.Could the authors clarify how the proposed ratio (number of times moderation is reported vs. total investigations) will be interpreted? Additionally, how will studies with incomplete or ambiguous reporting of moderation factors be handled?

Reviewer #2: Dear author/s,

Thank you for the opportunity to review this manuscript, which presents a protocol for a scoping review on promoting physical activity (PPA) among people with multiple sclerosis (MS). The protocol is well presented and provides a framework for an interesting and useful review. The search strategy, which includes the PRISMA-ScR checklist, appears to be comprehensive. My only concern is ensuring that the review does not extend beyond the limits of a scoping review. The research questions regarding evidence of moderation of the relations between factors that may qualify as a target for interventions and PPA among people with MS may lead to different synthesis methods. A scoping review will allow you to map these features as they are reported in the literature, but it is not an appropriate method for providing evidence on effectiveness or etiology.

If the main goal of the review is to map and characterize features of the reported literature on PPA among MS, then a scoping review is appropriate. However, I would suggest rephrasing the research questions slightly to align with the acronym PCC, which is more common for scoping reviews - Population, Concept, Context - as recommended in reference 27: Peters, M., Godfrey, C., McInerney, P., Khalil, H., Larsen, P., Marnie, C., Pollock, D., Tricco, A., Munn, Z. Best practice guidance and reporting items for the development of scoping review protocols. JBI Evidence Synthesis 20(4): p 953-968, April 2022. | DOI: 10.11124/JBIES-21-00242. If, however, the main goal is to determine effectiveness, then I suggest using systematic review methods, which will allow you to determine effectiveness, or a review of etiology and risk for identifying causal factors influencing outcomes.

These articles may be of use:

Munn, Z., Peters, M.D.J., Stern, C. et al. Systematic review or scoping review? Guidance for authors when choosing between a systematic or scoping review approach. BMC Med Res Methodol 18, 143 (2018). https://doi.org/10.1186/s12874- 018-0611-x

Tricco, A.C., Lillie, E., Zarin, W. et al. A scoping review on the conduct and reporting of scoping reviews. BMC Med Res Methodol 16, 15 (2016). https://doi.org/10.1186/s12874-016-0116-4

However, the feedback provided outlines several critical areas for improvement in the manuscript before it can be accepted for publication. Addressing these points will enhance the clarity and quality of the research presented.

1. The author should rewrite the abstract, because it does not adequately describe your work. The authors should ensure that it accurately reflects the scope and outcomes of their study.

2. The introduction is pivotal in setting the stage for the research. It should clearly articulate the main ideas and motivations behind the proposed study. The authors must explicitly state the research question and the motivation for their work, ensuring that the research gap is adequately addressed. This clarity will help readers understand the context and importance of the research. 3. Add a table of used symbols in the paper to improve readability. 4. The authors should comment on the relevance of the results, comparison of the approaches, and critical review of the relevant literature. 5. The authors should identify and discuss open problems related to their research. Highlighting these issues can provide a pathway for future research and demonstrate the ongoing relevance of their work.

6. The manuscript currently lacks sufficient literature citations. The authors should expand their discussion of related work.

7. It is crucial for authors to address potential biases or inconsistencies when reporting on moderating factors. This involves a systematic approach to ensure that the findings are credible and reflect the true nature of the relationships being studied.

8. The authors explain how to interpret the ratio of the number of times moderation is reported versus the total number of studies.

9. It seems that using seven databases and manual search to obtain the breadth of literature may not be enough. The comprehensiveness of the literature review is very important. Relying on only a limited number of databases can lead to gaps in the literature, as different databases may index different journals and articles. A systematic approach to literature search often involves the use of multiple databases, including specialized databases related to a particular field of study, to ensure a thorough exploration of available research.

**********

7. PLOS authors have the option to publish the peer review history of their article (what does this mean?). If published, this will include your full peer review and any attached files.

If you choose “no”, your identity will remain anonymous but your review may still be made public.

**Do you want your identity to be public for this peer review?**   For information about this choice, including consent withdrawal, please see our Privacy Policy.

Reviewer #1: No

Reviewer #2: No

**********

[NOTE: If reviewer comments were submitted as an attachment file, they will be attached to this email and accessible via the submission site. Please log into your account, locate the manuscript record, and check for the action link "View Attachments". If this link does not appear, there are no attachment files.]

While revising your submission, please upload your figure files to the Preflight Analysis and Conversion Engine (PACE) digital diagnostic tool, https://pacev2.apexcovantage.com/. PACE helps ensure that figures meet PLOS requirements. To use PACE, you must first register as a user. Registration is free. Then, login and navigate to the UPLOAD tab, where you will find detailed instructions on how to use the tool. If you encounter any issues or have any questions when using PACE, please email PLOS at figures@plos.org. Please note that Supporting Information files do not need this step.

# Author comment: Author response to Decision Letter 1

**DOI:** 10.1371/journal.pone.0351882.r002

31 Jan 2025

Our 'Response to Reviewers' document has been attached to this submission. Please see the document entitled: "Description of Revisions/Responses to Reviewers’ Comments PLOS ONE - Manuscript ID PONE-D-24-43698."

Attachment Submitted filename: Description of Revisions-1.31.2025-FINAL.docx

# Peer review: Decision Letter 1

**DOI:** 10.1371/journal.pone.0351882.r003

**Authors:** James Mockridge

2 Jun 2026

Potential intervention targets to promote physical activity among people with multiple sclerosis: A scoping review protocol for evidence of moderation

PONE-D-24-43698R1

Dear Dr. Amireault,

We’re pleased to inform you that your manuscript has been judged scientifically suitable for publication and will be formally accepted for publication once it meets all outstanding technical requirements.

Within one week, you’ll receive an e-mail detailing the required amendments. When these have been addressed, you’ll receive a formal acceptance letter and your manuscript will be scheduled for publication.

An invoice will be generated when your article is formally accepted. Please note, if your institution has a publishing partnership with PLOS and your article meets the relevant criteria, all or part of your publication costs will be covered. Please make sure your user information is up-to-date by logging into Editorial Manager at Editorial Manager® and clicking the ‘Update My Information' link at the top of the page. For questions related to billing, please contact billing support.

If your institution or institutions have a press office, please notify them about your upcoming paper to help maximize its impact. If they’ll be preparing press materials, please inform our press team as soon as possible -- no later than 48 hours after receiving the formal acceptance. Your manuscript will remain under strict press embargo until 2 pm Eastern Time on the date of publication. For more information, please contact onepress@plos.org.

Kind regards,

James Mockridge

Staff Editor

PLOS One

Additional Editor Comments (optional):

Reviewers' comments:

Reviewer's Responses to Questions

**Comments to the Author**

1. Does the manuscript provide a valid rationale for the proposed study, with clearly identified and justified research questions?

The research question outlined is expected to address a valid academic problem or topic and contribute to the base of knowledge in the field.

Reviewer #1: Yes

**********

2. Is the protocol technically sound and planned in a manner that will lead to a meaningful outcome and allow testing the stated hypotheses?

The manuscript should describe the methods in sufficient detail to prevent undisclosed flexibility in the experimental procedure or analysis pipeline, including sufficient outcome-neutral conditions (e.g. necessary controls, absence of floor or ceiling effects) to test the proposed hypotheses and a statistical power analysis where applicable. As there may be aspects of the methodology and analysis which can only be refined once the work is undertaken, authors should outline potential assumptions and explicitly describe what aspects of the proposed analyses, if any, are exploratory.

Reviewer #1: Yes

**********

3. Is the methodology feasible and described in sufficient detail to allow the work to be replicable?

Descriptions of methods and materials in the protocol should be reported in sufficient detail for another researcher to reproduce all experiments and analyses. The protocol should describe the appropriate controls, sample size calculations, and replication needed to ensure that the data are robust and reproducible.

Reviewer #1: Yes

**********

4. Have the authors described where all data underlying the findings will be made available when the study is complete?

The PLOS Data policy requires authors to make all data underlying the findings described in their manuscript fully available without restriction, with rare exception, at the time of publication. The data should be provided as part of the manuscript or its supporting information, or deposited to a public repository. For example, in addition to summary statistics, the data points behind means, medians and variance measures should be available. If there are restrictions on publicly sharing data—e.g. participant privacy or use of data from a third party—those must be specified.

Reviewer #1: Yes

**********

5. Is the manuscript presented in an intelligible fashion and written in standard English?

PLOS ONE does not copyedit accepted manuscripts, so the language in submitted articles must be clear, correct, and unambiguous. Any typographical or grammatical errors should be corrected at revision, so please note any specific errors here.

Reviewer #1: Yes

**********

6. Review Comments to the Author

Please use the space provided to explain your answers to the questions above and, if applicable, provide comments about issues authors must address before this protocol can be accepted for publication. You may also include additional comments for the author, including concerns about research or publication ethics.

You may also provide optional suggestions and comments to authors that they might find helpful in planning their study.

(Please upload your review as an attachment if it exceeds 20,000 characters)

Reviewer #1: Thank you for answering my question. I don't have any further inquiries at the moment. The authors may add response two to their study strengths.

**********

7. PLOS authors have the option to publish the peer review history of their article (what does this mean?). If published, this will include your full peer review and any attached files.

If you choose “no”, your identity will remain anonymous but your review may still be made public.

**Do you want your identity to be public for this peer review?**   For information about this choice, including consent withdrawal, please see our Privacy Policy.

Reviewer #1: No

**********

# Editor report: Acceptance letter

**DOI:** 10.1371/journal.pone.0351882.r004

**Authors:** James Mockridge

PONE-D-24-43698R1

PLOS One

Dear Dr. Amireault,

I'm pleased to inform you that your manuscript has been deemed suitable for publication in PLOS One. Congratulations! Your manuscript is now being handed over to our production team.

At this stage, our production department will prepare your paper for publication. This includes ensuring the following:

* All references, tables, and figures are properly cited

* All relevant supporting information is included in the manuscript submission,

* There are no issues that prevent the paper from being properly typeset

You will receive further instructions from the production team, including instructions on how to review your proof when it is ready. Please keep in mind that we are working through a large volume of accepted articles, so please give us a few days to review your paper and let you know the next and final steps.

Lastly, if your institution or institutions have a press office, please let them know about your upcoming paper now to help maximize its impact. If they'll be preparing press materials, please inform our press team within the next 48 hours. Your manuscript will remain under strict press embargo until 2 pm Eastern Time on the date of publication. For more information, please contact onepress@plos.org.

You will receive an invoice from PLOS for your publication fee after your manuscript has reached the completed accept phase. If you receive an email requesting payment before acceptance or for any other service, this may be a phishing scheme. Learn how to identify phishing emails and protect your accounts at https://explore.plos.org/phishing.

If we can help with anything else, please email us at customercare@plos.org.

Thank you for submitting your work to PLOS ONE and supporting open access.

Kind regards,

PLOS ONE Editorial Office Staff

on behalf of

Dr James Mockridge

Staff Editor

PLOS One
